# CAREGIVER IDENTITY IN EMERGING ADULTHOOD: HOW IDENTITY DISCREPANCY AND SALIENCE RELATE TO DISTRESS

**DOI:** 10.1093/geroni/igad104.3098

**Published:** 2023-12-21

**Authors:** Anastasia Canell, Grace Caskie

**Affiliations:** VA Bedford Healthcare System, Bedford, Massachusetts, United States; Lehigh University, Bethlehem, Pennsylvania, United States

## Abstract

As the older adult (age 65+) population grows in the United States, the demographics of caregivers are expanding to include more emerging adults (ages 18-25) who are participating in caregiving tasks. Minimal research has focused on this subgroup of caregivers to older adults, especially with regards to understanding factors that may influence their levels of caregiver distress. Guided by caregiver identity theory, the current study examined 1) the relationship between caregiver identity and caregiver distress, 2) support service utilization as a mediator between caregiver identity and caregiver distress, and 3) the inclusion of ageist attitudes within caregiver identity models. A sample of 259 emerging adult caregivers were recruited through Amazon MTurk. Fit of the hypothesized structural equation model was acceptable and squared multiple correlations (R2) indicated that 73.1% of the variance in caregiver distress, 23.8% of the variance in support service utilization, and 22.7% of the variance in ageist attitudes was explained by this model. Higher caregiver identity salience was significantly related to lower caregiver distress. Support service utilization partially mediated the relationship between identity salience and distress through an inconsistent mediation. The findings of the current study support caregiver identity as a unique, multidimensional process consisting of both identity discrepancy and salience latent constructs. Results showed low-to-moderate caregiver identity discrepancy, possibly due to identity flexibility during this developmental period. High caregiver identity salience was protective against caregiver distress for emerging adults. Ageist attitudes warrant inclusion in future research on caregiving identity development. Implications for support services are discussed.

